# ARTFit—A Quick and Reliable Tool for Performing Initial Fittings in Users of MED-EL Cochlear Implants

**DOI:** 10.3390/life12020269

**Published:** 2022-02-11

**Authors:** Lutz Gärtner, Timo Bräcker, Mathias Kals, Richard T. Penninger, Mareike Billinger-Finke, Thomas Lenarz, Andreas Büchner

**Affiliations:** 1 Department of Otolaryngology, Hannover Medical School, 30625 Hannover, Germany; lenarz.thomas@mh-hannover.de (T.L.); buechner.andreas@mh-hannover.de (A.B.); 2 MED-EL Research Center, MED-EL Elektromedizinische Geräte GmbH, 30625 Hannover, Germany; timo.braecker@medel.de; 3 MED-EL Elektromedizinische Geräte GmbH, 6020 Innsbruck, Austria; mathias.kals@medel.com (M.K.); richard.penninger@medel.com (R.T.P.); mareike.finke@medel.com (M.B.-F.)

**Keywords:** cochlear implant, electrically evoked compound action potential (ECAP), auditory nerve response telemetry (ART), ARTFit, automated fitting, speech perception

## Abstract

This study assessed the safety and performance of ARTFit, a new tool embedded in MAESTRO, the cochlear implant (CI) system software by MED-EL GmbH (Innsbruck, Austria). ARTFit automatically measures thresholds of the electrically evoked compound action potential (ECAP) to produce initial ‘maps’ (ECAPMAPs), i.e., configuration settings of the audio processor that the audiologist switches to live mode and adjusts for comfortable loudness (LiveECAPMAPs). Twenty-three adult and ten pediatric users of MED-EL CIs participated. The LiveECAPMAPs were compared to behavioral maps (LiveBurstMAPs) and to the participants’ everyday clinical maps (ClinMAPs). Four evaluation measures were considered: average deviations of the maximum comfortable loudness (MCL) levels of the LiveECAPMAPs and the LiveBurstMAPs from the MCLs of the ClinMAPs; correlations between the MCLs of the LiveECAPMAPs (MCL_ecap_) and the LiveBurstMAPs (MCL_burst_) with the MCLs of the ClinMAPs (MCL_clin_); fitting durations; and speech reception thresholds (SRTs). All evaluation measures were analyzed separately in the adult and pediatric subgroups. For all evaluation measures, the deviations of the LiveECAPMAPs from the ClinMAPs were not larger than those of the LiveBurstMAPs from the ClinMAPs. The Pearson correlation between the MCL_ecap_ and the MCL_clin_ across all channels was r^2^ = 0.732 (*p* < 0.001) in the adult and r^2^ = 0.616 (*p* < 0.001) in the pediatric subgroups. The mean fitting duration in minutes for the LiveECAPMAPs was significantly shorter than for that of the LiveBurstMAPs in both subgroups: adults took 5.70 (range 1.90–11.98) vs. 9.27 (6.83–14.72) min; children took 3.03 (1.97–4.22) vs. 7.35 (3.95–12.77). SRTs measured with the LiveECAPMAPs were non-inferior to those measured with the ClinMAPs and not statistically different to the SRTs measured with the LiveBurstMAPs. ARTFit is a safe, quick, and reliable tool for audiologists to produce ECAP-based initial fitting maps in adults and young children who are not able to provide subjective feedback.

## 1. Introduction

A cochlear implant (CI) is an auditory prosthesis used to restore hearing perception in people with severe to profound sensorineural hearing loss. Its multi-electrode array is normally inserted into the scala tympani and makes use of electrical stimulation to excite the surrounding neuronal population. In turn, this neuronal population generates action potentials that propagate along the auditory nerve and usually leads to auditory perception and speech comprehension.

Different surgical approaches are possible for CI implantation, e.g., [1]. At our clinic, a standard fashion as described in [2] is used.

A CI should be programmed—i.e., fitted—individually for each person using a CI. A fitting map is a set of electrical parameters that is adapted to each person’s individual needs with the goal being to achieve optimal sound perception. An initial fitting map is created during the first CI activation (when the audio processor is switched on). In the first few months after activation, several fitting sessions are usually necessary to refine the map, which then remains relatively stable over time [3].

Behavioral fitting procedures are currently considered standard clinical practice. One of the most important tasks in behavioral fitting is to determine the behavioral threshold (THR) and the level of maximum comfortable loudness (MCL) for each channel of the electrode array [4]. The THR and MCL define the lower and upper bounds of the electrical stimulation levels of each channel. The THR and MCL levels are measured by observing the response of the person using the CI to a series of short current bursts (i.e., biphasic charge-balanced electrical pulses) delivered to each channel, as in the implants manufactured by MED-EL GmbH (Innsbruck, Austria).

In the context of this paper, the set of parameters obtained from this fitting procedure is called a burst map (BurstMAP)—see Section 2.4 (Procedure) for details. When switched to live mode, a BurstMAP is usually not set to a satisfactory volume yet; the person using the CI might perceive sound as either too soft or too loud. Therefore, MCL levels should then be globally adjusted by scaling the MCL proportionally on all activated channels. This final map is called the LiveBurstMAP.

Another important task in behavioral fitting is balancing the perceived loudness of the channels along the electrode array. If single channels are set too loud or too soft compared to other channels, the person using the CI might adjust the global loudness by modifying the volume setting on their audio processor to prevent painful sensations or to compensate for a loss of sensitivity in certain frequency ranges. An imbalance in the loudness between channels can lead to reduced quality of hearing perception and poorer performance in speech perception tests [5,6]. In addition, an imbalance in the loudness between channels can affect the perception of spectral sound cues [7]. Thus, finding the right stimulation level for each channel and finding the right stimulation level across the entire electrode array are important and complex steps of the fitting procedure.

This behavioral approach to CI fitting is commonly used and is effective for many people. However, this approach can be very challenging in people who are not able to provide the audiologist with reliable (verbal) feedback, e.g., very young children and toddlers with limited expressive language skills [3,4], individuals with special needs, and the elderly [8]. In such cases, fitting cannot adequately be performed or completed, and the only option is to adopt a fitting procedure that is mainly based on the audiologist’s observation and subjective judgement [5].

Electrophysiological measures, such as the electrically evoked compound action potential (ECAP), electrically evoked stapedius reflex threshold, and electrically evoked auditory brainstem responses, are objective measures that have gained importance in these situations. Several studies investigated the quantitative properties of these objective measures and their potential clinical applications [9,10,11,12]. It has been suggested that these objective measures could be useful in producing reliable fitting maps that are not based on the subjective feedback of the person using the CI [13,14] and could possibly speed up the fitting process [4,5].

The ECAP is a measurement of the synchronized response of a group of auditory nerve fibers to an electrical stimulus [15]. Compared to other objective measures, there are several advantages to working with the ECAP: no extra equipment or external recording electrode is required to obtain an ECAP measurement [15]; intra-operative ECAP data remain sufficiently stable over time, which means they can be used postoperatively in initial fittings [16]; and, with the fitting software systems that are currently available, ECAP thresholds can be determined quickly and reliably [17,18,19]. The aforementioned characteristics make it easy to measure and collect ECAP data and to incorporate the ECAP as an objective measure into clinical practice.

Over the past two decades, several studies have assessed the extent to which ECAP measurements could be useful in fitting procedures [4,9,12,13,14,20,21]. High correspondence between visually determined ECAP thresholds (i.e., the audiologist’s observations) and automatically determined ECAP thresholds (by extrapolation from the amplitude growth function) have been found in several studies [17,18,22,23,24]. This suggests that ECAP thresholds that are automatically recorded by the CI’s system software could be a useful parameter in initial fittings, i.e., the fittings performed at activation or switch-on.

Some authors concluded that ECAP responses should be combined with subjective responses and that the initial fitting procedures should rely on both subjective and objective measures [21,25,26,27]. This recommendation is mainly due to large variations in the strength of the correlation between the ECAP threshold and the behavioral MCL levels that were measured by an audiologist. The correlation has been described as “poor”, “weak”, or “moderate” in both adult [25] and pediatric [28] study populations. Alvarez et al. [4] reported an “appreciable” statistically significant correlation between ECAP thresholds and the behavioral MCL levels. However, the authors concluded that ECAP thresholds should not be used to predict MCL levels; a fitting map based on ECAP thresholds alone would not be reliable. The relative error between the MCLs that were calculated from ECAP thresholds (with a linear regression model) and the behavioral MCLs (measured by an audiologist) was more than 20% for most channels. The authors cited a previous study that showed an estimation error ≥ 20% in the MCLs had significantly reduced the quality of hearing perception for the study participants [4,5]. Furthermore, He et al. [15] provided a comprehensive overview of ECAP studies that reported large variability in the between-subject and within-subject correlation in both the adult and pediatric study populations.

According to Vaerenberg et al. [3], there exist huge differences in clinical practice for fitting procedures. If there were concrete evidence on the reliability of an objective measure such as the ECAP, then the ECAP would become part of the common practices observed at CI centers. In 2012, a survey conducted at 47 CI centers in 17 countries found that 56% of the participating centers performed fittings by behavioral threshold determination. The remaining 44% used either intra- or post-operative ECAP thresholds to predict threshold stimulation values, and then proceeded to verify and adjust the thresholds behaviorally [3]. A 2018 systematic review of 37 publications that describe 32 studies on ECAP-based fitting procedures concluded that there is no substantial proof that ECAP data could be useful in fitting procedures. The authors concluded that the ECAP threshold is a weak predictor of both the behavioral threshold and of the MCL levels [8].

MAESTRO is the CI system software developed by MED-EL GmbH (Innsbruck, Austria). ARTFit is embedded in MAESTRO as an integrated software tool. ARTFit is based on AutoART [29], which uses FineGrain Technology to measure ECAP thresholds [23,30]. “FineGrain” is the name of a stimulation paradigm that samples the amplitude growth function in small stimulus steps, which allows for accurate ECAP threshold determination. Thus, ARTFit measures ECAP thresholds and then estimates the MCL levels on active channels along the electrode array. ECAP thresholds are recorded on adjacent electrodes at a sampling rate of 60 Hz. The user is provided with a visual indication of the measurement progress and if a threshold has been found. Blanking is used and an average of 14 curves are analyzed. These consist of seven anodic cathodic curves and seven cathodic anodic curves. System amplification is 100 times the baseline and the signal is 5 kHz low-pass filtered. An ECAP-based initial fitting map is produced, which the audiologist adjusts in live mode for comfortable loudness. 

The aim of this study was to assess ARTFit’s functionality in terms of four evaluation measures that were applied to the ECAP-based initial fitting maps produced with ARTFit: the average deviation of the MCLs per channel of the electrode array from the MCLs of clinical maps, correlations of the MCLs with those of clinical maps, fitting duration (FD), and speech reception threshold (SRT). The study provides a prospective and controlled assessment of the overall safety and performance of ARTFit in adult and pediatric users of MED-EL CIs.

## 2. Materials and Methods

### 2.1. ARTFit

MAESTRO offers ARTFit as an integrated tool for producing ECAP-based initial fitting maps. Within ARTFit, ECAP thresholds are automatically determined using MAESTRO’s AutoART function [29] using the FineGrain stimulation paradigm [30]. 

We used the default setting “Quick mode” throughout our study. In “Quick mode”, ECAP threshold determination occurs on at least four channels that are alternated between basal, medial, and apical sections along the electrode array. On channels where the ECAP threshold was impossible be determined, linear inter- and extrapolation are automatically applied to estimate the value of the threshold on those channels. Using the linear regression described in Alvarez et al. [4], normalized values for each channel are calculated. The normalized profile is thus “flattened”, as described in Alvarez et al. [4], and only 42% of the original ECAP threshold profile is present. Initial MCLs for all active channels are then calculated from the “flattened” normalized profile. This is done by scaling the charge level so that the maximum MCL in the initial map is 5 qu while preserving the MCL profile. If too few ECAP thresholds were found or were not spatially well-distributed, the initial MCLs on all active channels were set to the maximum of 5 qu.

The initial MCLs produced with ARTFit (ECAPMAP) are placed in MAESTRO’s fitting editor. The audiologist activates the map by switching it to live mode and globally adjusts the MCLs across all channels for comfortable loudness. This live adjusted map is the LiveECAPMAP that was stored on each participant’s audio processor.

### 2.2. Participants

Thirty-three participants were recruited and enrolled in the study: 23 adults (≥18 years) and 10 children (4–6 years). The inclusion criteria were as follows: all participants were users of uni- or bilateral MED-EL CIs who had at least 6 months of experience with implant use in the ear that was tested; participants’ clinical map should not have been based on ART thresholds; at least 10 out of the 12 channels (note that all CI electrode arrays by MED-EL have 12 channels) should have been active with the clinical map enabled in the ear to be tested; adult participants should be able to perform speech intelligibility tests with Hochmair–Schulz–Moser (HSM) sentences and achieve more than 35% at 10 dB signal-to-noise ratio (SNR), or an SRT of less than +5 dB in the Oldenburg Sentence Test for Children (OLKISA); pediatric participants should be able to perform an open-set speech test; and all participants should be fluent in German. The exclusion criteria were as follows: persons with a cochlear malformation who use a CI; and persons who use uni- and bilateral CIs with electric-acoustic stimulation (EAS). 

#### 2.2.1. Adult Subgroup

Twenty-three adult participants were enrolled in the study. One adult participant (A01) was withdrawn from the study and excluded from all statistical analyses due to a protocol deviation. Data for 22 adult participants (8 males, 14 females) were included in the statistical analyses. The mean age at enrolment was 60.2 years (±11.9 years SD; range 32.1 years to 77.5 years). The mean age at implantation was 55.5 years (±12.7 years SD; range 19.5 years to 75.3 years). The adult participants were implanted with Sonata TI100, Pulsar CI100, Concerto, or Synchrony, and with a Standard, FLEXSOFT, FLEX28, or FLEX24 electrode array. An OPUS 2, OPUS 2XS, SONNET, or RONDO audio processor was in use.

Table 1 summarizes the demographic data of the adult subgroup.

#### 2.2.2. Pediatric Subgroup

Ten pediatric participants (9 males, 1 female) were enrolled in the study. One pediatric participant (C04) was withdrawn from the study as per the decision of his/her parent. After fitting procedures were completed, there was a long waiting time before we could proceed with the speech perception tests. This imposed too much stress on this participant. Therefore, he/she was included in the demographic data but excluded from all statistical analyses. Data for two pediatric participants (C02 and C08) were incomplete and the participants were excluded from the respective analyses (see Section 3). The mean age at enrollment was 5.7 years (±0.89 SD; range 4.0–6.8 years). The mean age at implantation was 15.4 months (±10.6 SD; range 5.9–39.2 months). The pediatric participants were implanted with Concerto or Synchrony, and with a FLEX28, FLEX24, or FLEX20 electrode array. An OPUS 2 or a SONNET audio processor was in use.

Table 2 summarizes demographic data on the pediatric subgroup.

### 2.3. Study Design

This clinical investigation was a prospective, acute, monocentric study. All study participants were tested according to the same study procedures and had only one appointment at the ENT Department for the purpose of this study. This appointment was scheduled in addition to the study participants’ routine visits. The participants were asked on the same day if they would be willing to participate in the study. The participants were not compensated for their participation in the study—only travel expenses were reimbursed.

### 2.4. Procedure

Our participants were either (1) bilateral CI users or (2) unilateral CI users with very little functional residual hearing on the contralateral side. In order to avoid bilateral benefit, the contralateral ear of participants with unilateral CIs was plugged throughout the fitting procedure and during the speech perception tests. The better performing ear of participants using bilateral CIs was determined by looking at the clinic’s records of their speech perception test results. This ear was tested throughout the study.

Impedance field telemetry (IFT) measurements were performed first. By measuring impedances on all electrodes, it was determined whether each electrode along the array was working properly. Only participants with at least ten active electrodes could proceed with the study; this was a requirement to participate in this study. All candidates fulfilled this requirement and no candidate had to be excluded for this reason. 

The map with which participants entered the study was saved and used as their ClinMAP. MED-EL recommends that THR levels are set to 10% of the MCLs on all active channels. This is the recommended default setting for THR levels since MAESTRO 7.0. All study participants use MED-EL CIs, thus THR levels on all active channels remained at this default setting in the ClinMAPs. A default volume of 90% was used, allowing users to increase the volume until 100% in soft listening conditions. If deemed necessary, adjustments to the ClinMAPs were made based on participants’ subjective feedback, according to standard clinical practice. In the pediatric subgroup, adjustments were made on an individual basis according to the clinic’s internal guidelines, while taking each pediatric participant’s developmental status into account. These adjustments were incorporated into the ClinMAPs before the start of any study-related measurements.

In the adult subgroup, maps based on behavioral fitting procedures (BurstMAPs) were produced as follows. A new map with default settings was produced. In behavioral methods, MCLs are usually measured on all activated channels before setting the corresponding THR levels on those channels. Thus, the MCLs were measured with fitting bursts (burst MCLs) on each channel first and then the corresponding THR levels were set to 10% of the burst MCL on each channel. During the final step, the perceived loudness across all channels was balanced relative to the loudness of a medially located channel, mostly E06. 

In the pediatric subgroup, burst MCLs were measured in the same way as in the adult subgroup. The corresponding THR levels were then set to 10% of the burst MCL on each channel.

In both subgroups, the MCLs of the BurstMAPs were globally adjusted in live mode according to the participants’ subjective feedback. The resultant maps of this procedure were live adjusted BurstMAPs, i.e., the LiveBurstMAPs. The fitting duration of the LiveBurstMAPs (FD_burst_) refers to the time required to carry out the procedures that produce the LiveBurstMAPs, beginning with bursting the first channel of a default map (BurstMAP), oversetting of MCLs, balancing, and final global adjustment of loudness in live mode. This duration was recorded with a stopwatch. Both the LiveBurstMAPs and ECAP-based maps were produced during the participants’ single test appointment. To ensure that the LiveBurstMAPs were produced independently of and remained unbiased by the ECAP-based procedure, the LiveBurstMAPs were always produced first and the ECAP-based maps thereafter.

ECAP-based maps (ECAPMAPs) were produced with ARTFit as follows. A map with the default settings was produced with MAESTRO’s ARTFit workflow. Channels that were disabled in each participant’s ClinMAP were also disabled for their ECAPMAP. As in the fitting procedure used to produce the BurstMAPs, THR levels were set to 10% of the ECAP-based MCLs on each channel. A default volume of 90% was used. Once the audiologist switched the map to live mode, MCLs were globally adjusted according to the participants’ subjective feedback. The resultant maps of this procedure were the live adjusted ECAPMAPs, i.e., the LiveECAPMAPs. The fitting duration of the LiveECAPMAPs (FD_ecap_) refers to the time required to carry out the procedures that produce the LiveECAPMAPs, beginning with the ARTFit workflow, over the ECAP measurements and final global adjustments in live mode. This duration was recorded with a stopwatch.

The three maps (ClinMAP, LiveBurstMAP, and LiveECAPMAP) were saved on program locations 1, 2, and 3 of the participants’ audio processors, and were later used in the speech perception tests. The order in which the maps were saved on the three program locations was pseudo-randomized (according to the individual participant’s study file) to reduce biased learning effects on the study results. A copy of each participant’s ClinMAP was saved on program location 4. The audiologist who conducted the fitting also programmed the audio processor. The audiologist who conducted the speech perception tests did not know to which program locations the first three maps were saved. The participants were not told in which program location different maps were saved. See the following section, Evaluation measures, for how the study hypotheses were formulated.

Speech perception was assessed with OLKISA, which is an adaptation of the Oldenburg Sentence Test (OLSA). It is a validated and reliable speech audiometry test that is suitable for any age ≥ 4 years [31]. OLKISA was chosen to ensure that the speech reception threshold (SRT) scores of the adult and pediatric subgroups were comparable. The value of the SRT, measured in dB, where 50% of the words presented in a sentence were correctly understood (SRT50), was assessed. A constant speech level at 65 dB SPL and adaptive speech-shaped noise were presented to participants from 0° azimuth. In an adaptive procedure, the noise level was adapted until SRT50 was found [32]. To minimize reverberations and disturbances from outside, the speech tests were performed in a sound treated room. 

The participants had a training phase before the speech perception test phase. The adult participants were given two training lists and the pediatric participants were given only one training list. All participants used their ClinMAP during the training phase. As already mentioned earlier, the ClinMAPs were stored on program location 4, which was only used for this purpose. The training phase(s) started at +20 dB SNR. The results of the training phase(s) were used to determine the starting SNR of the test phases by adding +10 dB to each participant’s (average) SRT. In this way, comparable initial conditions for the adaptive test were created.

Similarly, during the test phase, the adult participants were tested with two test lists and the pediatric participants were tested with only one test list. This was the case for each of the three maps saved on their audio processor, i.e., the ClinMAP, LiveBurstMAP, and LiveECAPMAP, which were randomly saved on program locations 1, 2, and 3. 

### 2.5. Evaluation Measures

Deviations to ClinMAP MCLs in any direction, namely too high or too low, can be expected to be detrimental for the CI user. Consequently, the absolute value of the deviations is of interest in this analysis. As the clinical MAP is expected to be the most detailed, any new map has to be compared with it. To interpret the amount that the absolute difference is, in relation to ClinMAP MCLs, is relevant. Therefore, for the the comparison of MCLs per channel for all the active channels of the electrode array for each of the participants’ LiveECAPMAP and LiveBurstMAP, the following quantities were defined:

1. For any given channel, RD_ecapMCL_ is the magnitude of the relative difference (in %) between the MCL of the LiveECAPMAP (MCL_ecap_) and the MCL of the ClinMAP (MCL_clin_), with MCL_clin_ as the reference value.

(1) $${\mathrm{RD}}_{\mathrm{ecapMCL}}=\left|\;\frac{{\mathrm{MCL}}_{\mathrm{clin}}-{\;\mathrm{MCL}}_{\mathrm{ecap}}}{{\mathrm{MCL}}_{\mathrm{clin}}}\;\right|*100$$

2. For any given channel, RD_burstMCL_ is the magnitude of the relative difference (in %) between the MCL of the LiveBurstMAP (MCL_burst_) and the MCL of the ClinMAP (MCL_clin_), with MCL_clin_ as the reference value.

(2) $${\mathrm{RD}}_{\mathrm{burstMCL}}=\left|\;\frac{{\mathrm{MCL}}_{\mathrm{clin}}-{\;\mathrm{MCL}}_{\mathrm{burst}}}{{\mathrm{MCL}}_{\mathrm{clin}}}\;\right|*100$$

The quantities RD_ecapMCL_ and RD_burstMCL_ were compared in the adult subgroup. It was hypothesized that the difference, Δ_MCL_, between RD_ecapMCL_ and RD_burstMCL_ would be less than 20%. Therefore, the following hypothesis was tested:

ΔMCL = RDecapMCL − RDburstMCL < 20%(3)

**Figure 1:**
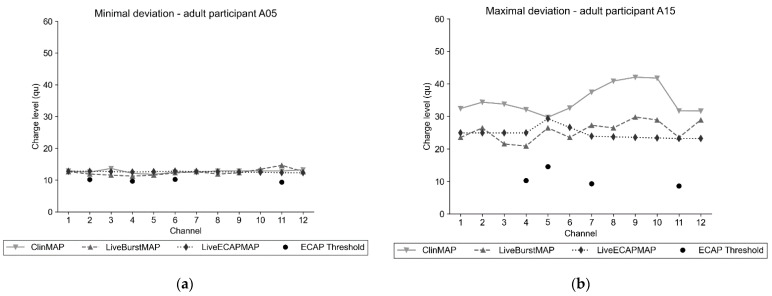

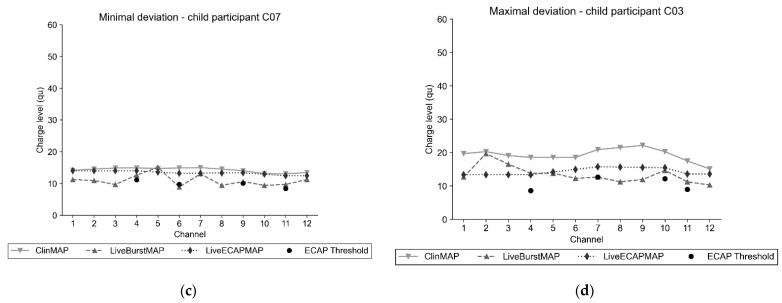
Charge level per channel for selected study participants. The charge level per channel of four participants for each map was saved on their audio processors. The ECAP threshold (solid dot) used to calculate the LiveECAPMAPs (diamond) is shown. Recall that ARTFit’s default setting ‘Quick mode’ was used throughout the study. In ‘Quick mode’, at least four channels are measured in an order that scans the entire electrode array. Thus, only four measurements of the ECAP threshold are shown for each participant. Adult subgroup: (**a**) the minimal deviation of the LiveECAPMAP from the ClinMAP was observed in participant A05; (**b**) the maximal deviation of the LiveECAPMAP from the ClinMAP was observed in participant A15. Pediatric subgroup: (**c**) minimum and (**d**) maximum deviations of the LiveECAPMAP from the ClinMAP were observed in participants C07 and C03, respectively.

The above criterion was based on the work of Sainz et al. [5], whereby imbalances in both the THR levels and the MCL levels have negative effects on the hearing sensitivity of the person using the CI. However, an imbalance in the MCL levels has a greater negative effect on hearing sensitivity than an imbalance in THR levels. Therefore, an imbalance in the MCL levels is of greater clinical importance than an imbalance in the THR levels. An underestimation of the MCL level in one channel does not affect the remaining balanced channels but reduces hearing sensitivity in the frequency band associated with the unbalanced channel. The authors provided a quantitative evaluation of the tolerance for an imbalance in MCL levels: an underestimation of 20% in the MCL levels reduces hearing sensitivity significantly, i.e., the non-overlapping confidence interval, by about 11 dB hearing loss (HL). Hence, we extrapolated from Sainz et al. [5] (Figure 1) that a difference of <20% is not of clinical relevance.

To compare the average deviation of the LiveECAPMAPs from the ClinMAPs with the average deviation of the LiveBurstMAPs from the ClinMAPs, the following quantities were defined:

3. RD_ecapMAP_ is the mean of RD_ecapMCL_ (Equation (1)).
4. RD_burstMAP_ is the mean of RD_burstMCL_ (Equation (2)).

The quantities RD_ecapMAP_ and RD_burstMAP_ were compared in the adult subgroup. It was hypothesized that the difference, Δ_MAP_, between RD_ecapMAP_ and RD_burstMAP_ would be less than 20%, thereby testing the following hypothesis:

Δ_MAP_ = RD_ecapMAP_ − RD_burstMAP_ < 20% (4)

To assess if the ECAP-based maps produced with ARTFit could save time in the clinical setting, the mean percentage difference in the fitting duration of the maps was considered: the fitting duration FD (in minutes) of the LiveECAPMAPs was compared to the FD (in minutes) of the LiveBurstMAPs. It was hypothesized that, in the adult subgroup, the mean fitting duration of the LiveECAPMAPs (FD_ecap_) would be shorter than the mean fitting duration of the LiveBurstMAPs (FD_burst_). A superiority margin was set for the mean percentage difference, Δ_FD_ (in %), in the fitting duration of the two fitting maps, with FD_burst_ as the reference value:

(5) $${∆}_{\mathrm{FD}}=100*\;\frac{\left({\mathrm{FD}}_{\mathrm{burst}}-{\;\mathrm{FD}}_{\mathrm{ecap}}\right)}{{\mathrm{FD}}_{\mathrm{burst}}}>10\%$$

We assumed that an FD_ecap_ that is shorter than FD_burst_ by 10% is clinically relevant.

To evaluate the quality of each map, participants’ speech reception threshold (SRT) was measured. The relative difference to the ClinMAP is used, since large SRT variations in the ClinMAP across subjects can be expected and are not the focus of our analysis. The following quantities were used:

RD_ecapSRT_ is the difference (in dB) between the SRTs that were measured when participants were fitted with the LiveECAPMAPs (SRT_ecap_) and the SRTs that were measured when participants were fitted with their ClinMAPs (SRT_clin_).

RD_ecapSRT_ = SRT_ecap_ − SRT_clin_, (6)

5. RD_burstSRT_ is the difference (in dB) between the SRTs that were measured when participants were fitted with the LiveBurstMAPs (SRT_burst_) and the SRTs that were measured when participants were fitted with their ClinMAPs (SRT_clin_).

RD_burstSRT_ = SRT_burst_ − SRT_clin_ (7)

In accordance to Article 21(4) of the Assistive Technology Guideline of the Federal Joint Committee [33], a difference in SRT ≥ 2 dB was regarded as clinically significant. Thus, in order to compare RD_ecapSRT_ and RD_burstSRT_ in the adult subgroup, it was hypothesized that the difference, Δ_SRT_, between RD_ecapSRT_ and RD_burstSRT_ would be less than 2 dB. The following hypothesis was tested:

Δ_SRT_ = RD_ecapSRT_ − RD_burstSRT_ < 2 dB, (8)

The same set of quantities defined in Equations (3), (4), (5), and (8) were measured in the pediatric subgroup. As long as a paired *t*-test is used, the SRT_ecap_ and the SRT_burst_ can also be directly compared. The data obtained in the pediatric subgroup were separately evaluated in an explorative analysis of the subgroup.

### 2.6. Statistical Methods

The one-sample inference test was used for the sample size calculations (G*Power 3.1). A minimum sample size of 22 participants was calculated for the primary endpoint and for the co-primary endpoint. For the primary endpoint (∆_MAP_), a non-inferiority margin of 20% and a standard deviation (±SD) of 37.63% [4] with a power of 80% and an alpha-level of 0.05 was used. For the co-primary endpoint (∆_FD_), a non-inferiority margin of 1.17 min that is 10% of the standard fitting method for LiveBurstMAPs and a standard deviation (±SD) of 2.16 min [34] with a power of 80% and an alpha-level of 0.05 was used. Due to the exploratory nature of the analysis in the pediatric subgroup, no sample size calculation was possible for this subgroup. The paired sample *t*-test or the Wilcoxon signed-rank test were used for pairwise comparisons. To verify the data distribution, the Kolmogorov–Smirnov test together with the Shapiro–Wilk test were applied. Statistical significance was set to *p* ≤ 0.05. IBM SPSS Statistics Version 24 (IBM, Armonk, New York, NY, USA) was used for the analyses. Figures were plotted in R.

## 3. Results

The study objectives and hypotheses were formulated for the adult subgroup and extrapolated to the pediatric subgroup. The same set of measures were used and separately analyzed in the two subgroups.

### 3.1. Charge Levels per Channel

Figure 1 shows the charge levels per channel of each map (ClinMAP, LiveBurstMAP, LiveECAPMAP) that was saved to the participants’ audio processors and the ECAP thresholds used to produce the LiveECAPMAPs. The charge levels of two adult and two pediatric participants are shown. Figure 1a shows the adult participant, A05, who had the minimal (i.e., the smallest) deviation of their LiveECAPMAP from their ClinMAP. Figure 1b shows the adult participant, A15, who had the maximal (i.e., largest) deviation in those two maps. Similarly, Figs 1c and 1d show the charge levels per channel of each map and the ECAP thresholds for the pediatric participants who had the minimal and maximal deviations of their LiveECAPMAPs from their ClinMAPs. In 24 of 31 participants, the ECAP thresholds were lower than the charge levels of all three maps.

It was observed in both subgroups that the MCL levels of the ECAP-based maps before activation, i.e., the ECAPMAPs, were lower on each channel than the MCL levels of the ClinMAP on the corresponding channel. This gave the audiologist an indication that, before switching to live mode, the ECAP-based maps were not uncomfortably loud for the person using the CI.

**Figure 2:**
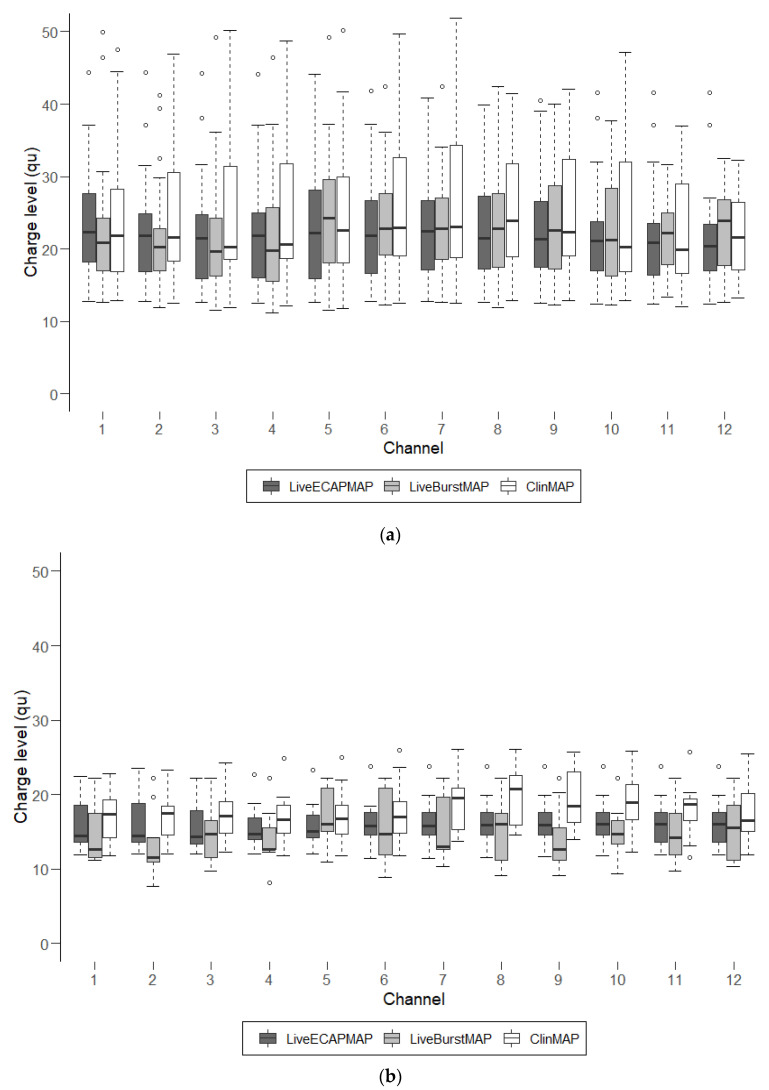
Charge levels of the three maps for the two subgroups. The charge level per channel taken over all participants in (**a**) the adult subgroup and (**b**) the pediatric subgroup for each map saved to participants’ audio processor. Median values are shown as black horizontal lines. Open circles represent outliers. The length of the box represents the interquartile range (IQR). The bottom edge of the box is the 25th percentile (lower quartile) and the top of the box is the 75th percentile (upper quartile); the median (thick horizontal line) is the 50th percentile. The length of a whisker corresponds to data points and extends to a maximum of 1.5 times the IQR. Outliers are defined as data points that lie 1.5 times the box height above (below) the 75th (25th) percentile.

Figure 2 shows the charge levels for all three maps in the adult (Figure 2a) and pediatric (Figure 2b) subgroups. The relationship between the maps observed in the four participants shown in Figure 1 seems to be representative of both subgroups. In both the adult and pediatric subgroups, the ClinMAPs lie above the LiveBurstMAPs and LiveECAPMAPs; whereas no consistent pattern could be identified between the LiveBurstMAPs and LiveECAPMAPs.

A difference between the two subgroups could be discerned from Figure 2. It was observed that the MCL levels across the three maps are, in general, higher in the adult subgroup than in the pediatric subgroup. Both subgroups had one test session, but children are usually fitted over several sessions. It was a challenging task to perform fittings in the pediatric subgroup in one test session. 

### 3.2. Average Deviation of MCLs per Channel

The relative difference, RD_ecapMCL_, between the MCLs of the LiveECAPMAPs (MCL_ecap_) and the MCLs of the participants’ ClinMAPs (MCL_clin_) was compared to the relative difference, RD_burstMCL_, between the MCLs of the LiveBurstMAPs (MCL_burst_) and the MCLs of the participants’ ClinMAPs (MCL_clin_) in the adult subgroup. It was hypothesized that their difference, Δ_MCL_ = (RD_ecapMCL_ − RD_burstMCL_), would be less than 20% for each channel (see Equation (3)).

#### 3.2.1. Adult Subgroup

**Figure 3:**
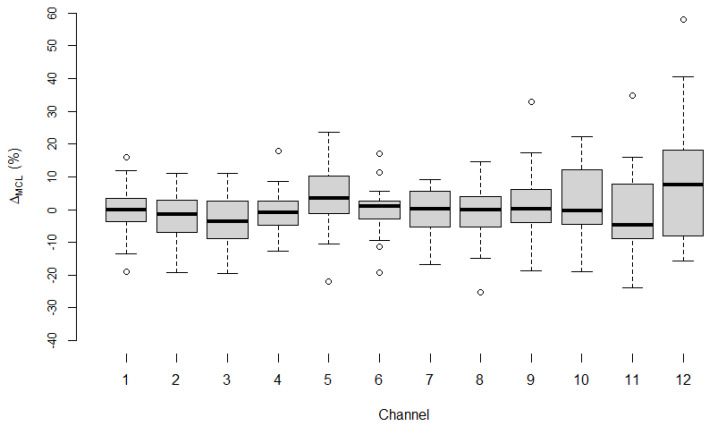
Δ_MCL_ for all 12 channels in the adult subgroup. The difference, Δ_MCL_, between RD_ecapMCL_ and RD_burstMCL_ per channel in the adult subgroup. Median values are shown as black horizontal lines. Open circles represent outliers. See the caption of Figure 2 on how to interpret the box-and-whisker plots.

Figure 3 shows Δ_MCL_ for all 12 channels in the adult subgroup. The mean of Δ_MCL_ was calculated for each of the 12 channels and ranged between −2.84% and +8.77%.

For all 12 channels, the values of RD_ecapMCL_ and RD_burstMCL_ did not differ significantly (Wilcoxon signed-rank test: *p*-values for the Δ_MCL_ per channel ranged from 0.131 to 0.904). The 95% confidence interval (c.i.) across all channels had an upper bound that ranged from +0.87% to +19.56% and a lower bound that ranged from −7.26% to −1.93%. Therefore, the 95% confidence interval supported the hypothesized non-inferiority margin of <20% for all channels.

There is a high group correlation, i.e., taken over the participants of the adult subgroup, between the MCL_ecap_ and MCL_clin_ values across all channels (r^2^ = 0.732; *p* < 0.001). A similar group correlation between the MCL_burst_ and MCL_clin_ values (r^2^ = 0.779; *p* < 0.001) across all channels was found. The group correlation between the ECAP thresholds and the MCL_clin_ across all channels was, in contrast, relatively poor (r^2^ = 0.149; *p* < 0.001).

#### 3.2.2. Pediatric Subgroup

**Figure 4:**
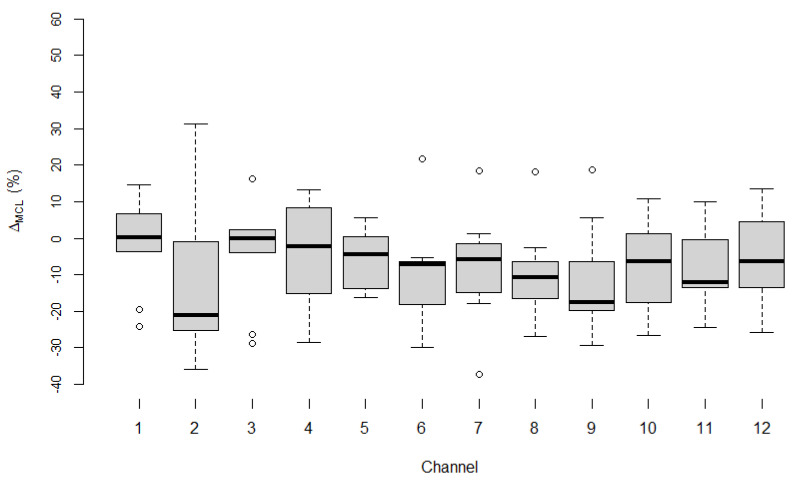
Δ_MCL_ for all 12 channels in the pediatric subgroup. The difference, Δ_MCL_, between RD_ecapMCL_ and RD_burstMCL_ per channel in the pediatric subgroup. Median values per channel are shown as black horizontal lines. Open circles represent outliers.

Figure 4 shows Δ_MCL_ for all 12 channels in the pediatric subgroup. The mean of Δ_MCL_ for each channel was calculated in the same way as for the adult subgroup and ranged from −12.32% to −1.43%.

RD_ecapMCL_ and RD_burstMCL_ across all channels did not differ significantly (Wilcoxon signed-rank test: *p*-values for Δ_MCL_ per channel ranged from 0.066 to 0.953). The 95% confidence interval had an upper bound that ranged from −0.25% to 8.47% and a lower bound that ranged from −28.56% to −11.32% across all channels.

As in the case of the adult subgroup, a high group correlation was found between the MCL_ecap_ and the MCL_clin_ values across all channels (r^2^ = 0.616; *p* < 0.001). A lower group correlation between the MCL_burst_ and the MCL_clin_ values across all channels (r^2^ = 0.517; *p* < 0.001) was found. The group correlation between the ECAP thresholds and MCL_clin_ across all channels was poor and statistically not significant (r^2^ = 0.001; *p* = 0.880).

### 3.3. Average Deviation of the Fitting Maps

The relative difference, RD_ecapMAP_, between the LiveECAPMAPs and the participants’ ClinMAPs was compared to the relative difference, RD_burstMAP_, between the LiveBurstMAPs and the participants’ ClinMAPs in the adult subgroup. It was hypothesized that the difference, Δ_MAP_, between these two quantities, i.e., (RD_ecapMAP_ − RD_burstMAP_), is less than 20% (see Equation (4)). 

#### 3.3.1. Adult Subgroup

**Figure 5:**
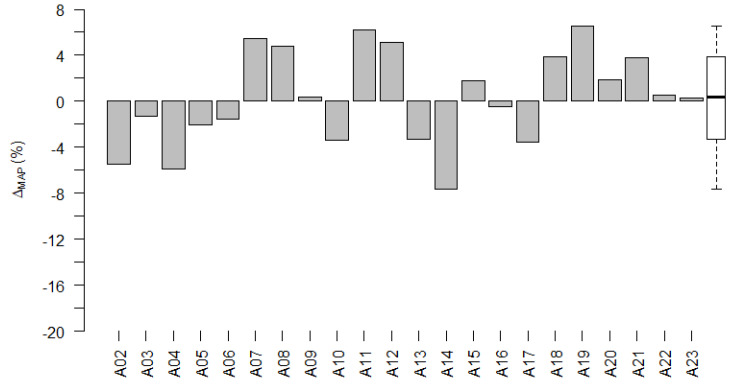
Δ_MAP_ per adult participant. The difference, Δ_MAP_, between RD_ecapMAP_ and RD_burstMAP_ for participants of the adult subgroup. The group data of Δ_MAP_ are shown as a box plot to the right, where the median value is shown as a black horizontal line.

Figure 5 shows Δ_MAP_ for each participant of the adult subgroup. For all participants, Δ_MAP_ lies between ± 8%. For 10 out of the 22 participants, Δ_MAP_ was negative (A02, A03, A04, etc.). In these 10 participants, the LiveECAPMAPs differed less from their ClinMAPs than the LiveBurstMAPs differed from their ClinMAPs. In other words, these 10 participants’ LiveECAPMAPs were more similar to their ClinMAPs than the LiveBurstMAPs were to their ClinMAPs. In the other 12 participants, their LiveBurstMAPs were more similar to their ClinMAPs than their LiveECAPMAPs.

The values of RD_ecapMAP_ and RD_burstMAP_ did not differ significantly (Wilcoxon signed-rank test: *p* = 0.590). The mean of Δ_MAP_ was 0.47% (95% c.i. −1.43% to +2.36%; range: −7.60% to +6.57%). Thus, the 95% confidence interval supported the hypothesis for non-inferiority of the LiveECAPMAPs to the LiveBurstMAPs.

#### 3.3.2. Pediatric Subgroup

**Figure 6:**
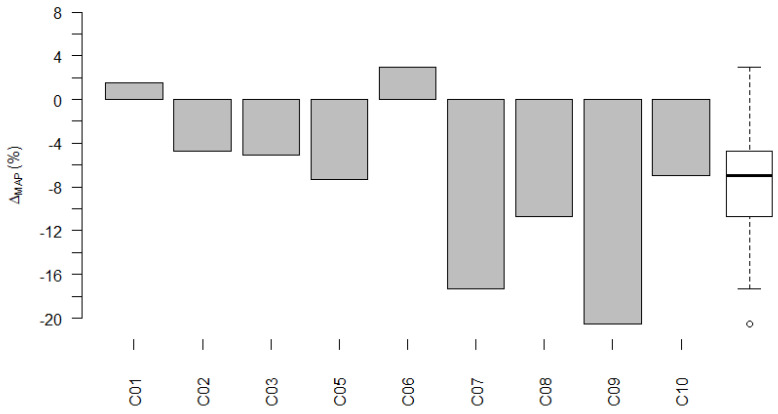
Δ_MAP_ per pediatric participant. The difference, Δ_MAP_, between RD_ecapMAP_ and RD_burstMAP_ for participants of the pediatric subgroup. The group data of Δ_MAP_ are shown as a box plot to the right, where the median value is shown as a black horizontal line.

Figure 6 shows Δ_MAP_ for the pediatric subgroup. For seven of the nine participants, Δ_MAP_ was negative. For these participants, the LiveECAPMAPs differed less from their ClinMAPs than the LiveBurstMAPs differed from their ClinMAPs (i.e., the LiveECAPMAPs were more similar to the ClinMAPs than the LiveBurstMAPs were to the ClinMAPs). RD_ecapMAP_ and RD_burstMAP_ did differ significantly (Wilcoxon signed-rank test: *p* = 0.021). The mean of Δ_MAP_ was −7.57% (95% c.i. −1.61% to +13.52%; range: −20.5% to +2.99%).

### 3.4. Fitting Duration

The fitting duration of the LiveECAPMAPs (FD_ecap_) was compared to the fitting duration of the LiveBurstMAPs (FD_burst_) in the adult subgroup. It was hypothesized that the mean of FD_ecap_ (in minutes) is shorter than the mean of FD_burst_. Furthermore, a Δ_FD_ of −10%, as in Equation (5), was considered clinically relevant.

#### 3.4.1. Adult Subgroup

**Figure 7:**
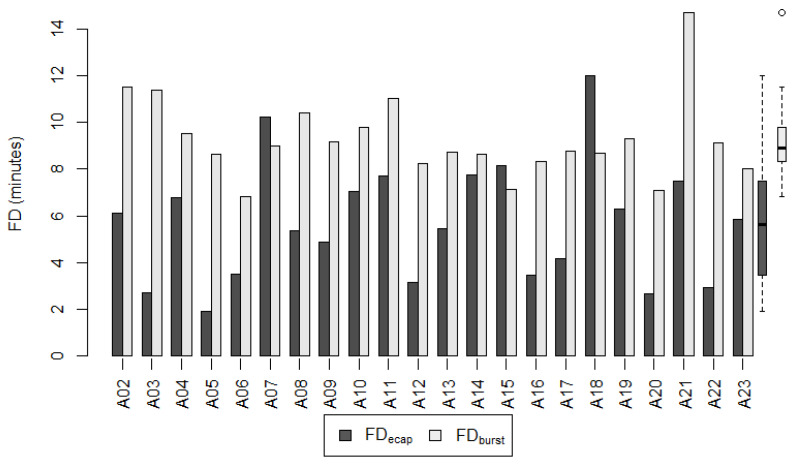
Fitting duration of the live adjusted maps per adult participant. Fitting duration FD_ecap_ for the LiveECAPMAPs (dark grey) and FD_burst_ for the LiveBurstMAPs (light grey) in the adult subgroup. The group data of FD_ecap_ and FD_burst_ are shown as boxplots to the right, where the median values are shown as black horizontal lines.

Figure 7 shows FD_ecap_ and FD_burst_ for each participant of the adult subgroup. The mean of FD_ecap_ was significantly lower than the mean of FD_burst_ (Wilcoxon signed-rank test: *p* < 0.001). On average, the fitting duration with FD_ecap_ was 37.5% (± SD: 29.75%; 95% CI: −50.71% to −24.32%) shorter compared to FD_burst_. The mean of FD_ecap_ was 5.70 ± 2.59 min (range 1.90 min to 11.98 min) and the mean of FD_burst_ was 9.27 ± 1.75 min (range 6.83 min to 14.72 min). 

The 95% confidence interval supported the hypothesized superiority of the LiveECAPMAPs over the LiveBurstMAPs in terms of the fitting duration.

#### 3.4.2. Pediatric Subgroup

**Figure 8:**
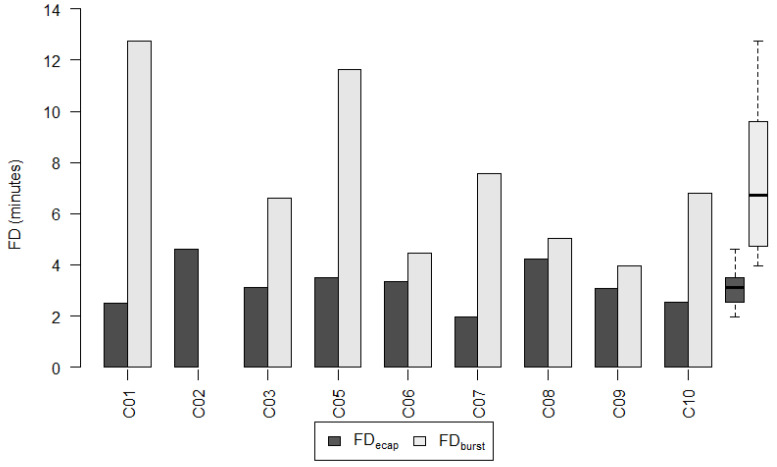
Fitting duration of the live adjusted maps per pediatric participant. Fitting duration FD_ecap_ for the LiveECAPMAPs (dark grey) and FD_burst_ for the LiveBurstMAPs (light grey) in the pediatric subgroup. For participant C02, a flat map was fitted instead of a LiveBurstMAP and, therefore, a comparison of its fitting duration with that of the LiveECAPMAP cannot be shown. The group data of FD_ecap_ and FD_burst_ are shown as boxplots to the right, where the median values are shown as solid black horizontal lines.

Figure 8 shows the fitting duration for the pediatric subgroup. The fitting of young children is a challenge that must be met with patience. We did not succeed in establishing a LiveBurstMAP for participant C02. A flat map was fitted instead, i.e., all MCLs were set to the same value and no live adjustments were made. A comparison of the fitting duration of a LiveBurstMAP to the fitting duration of a LiveECAPMAP cannot be shown for this participant. The participant was excluded from this analysis.

The mean of FD_ecap_ was significantly lower than the mean of FD_burst_ (Paired sample *t*-test: *p* = 0.010). On average, the fitting duration with FD_ecap_ was 50.4% (±SD: 25.59%; 95% CI: −71.80% to −29.01%) shorter compared to FD_burst_. The mean of FD_ecap_ was 3.03 ± 0.69 min (range 1.97–4.22 min) and the mean of FD_burst_ was 7.35 ± 3.25 min (range (3.95–12.77 min). 

### 3.5. Speech Reception Threshold

The difference, RD_ecapSRT_, between the SRT_ecap_ and the SRT_clin_ was compared to the difference, RD_burstSRT_, between the SRT_burst_ and the SRT_clin_. It was hypothesized that their difference, Δ_SRT_, is less than 2 dB (see Equation (8)).

#### 3.5.1. Adult Subgroup

**Figure 9:**
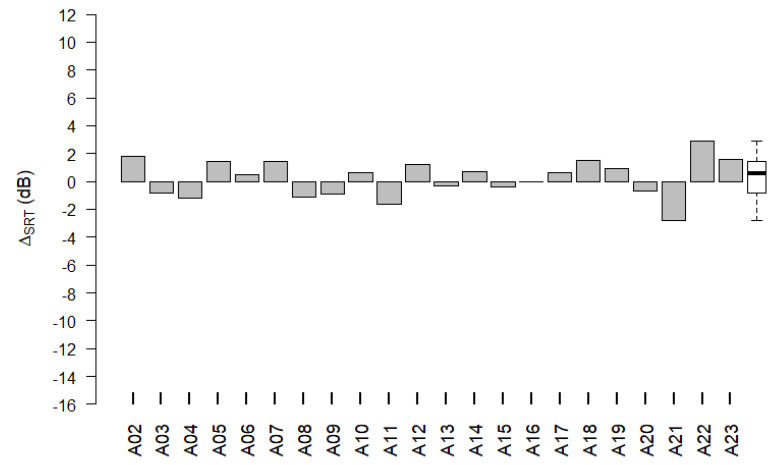
Δ_SRT_ per adult participant. The difference, Δ_SRT_, between RD_ecapSRT_ and RD_burstSRT_ for participants in the adult subgroup. Group data of Δ_SRT_ are shown as a boxplot to the right, where the median value is shown as a black horizontal line.

Figure 9 shows Δ_SRT_ for each participant of the adult subgroup. The SRTs used in the data analysis were calculated by averaging the results from conducting the speech perception tests with two OLKISA test lists. The values of RD_ecapSRT_ and RD_burstSRT_ did not differ significantly (paired sample *t*-test: *p* = 0.404). For 20 of the 22 participants, the absolute value of Δ_SRT_ was less than 2 dB. For the remaining two participants (A21 and A22), the absolute value of Δ_SRT_ was greater than 2 dB. The mean Δ_SRT_ was 0.25 dB (95% c.i. −0.35 dB to +0.85 dB) in the adult subgroup, which supported the hypothesized non-inferiority margin of <2 dB.

#### 3.5.2. Pediatric Subgroup

**Figure 10:**
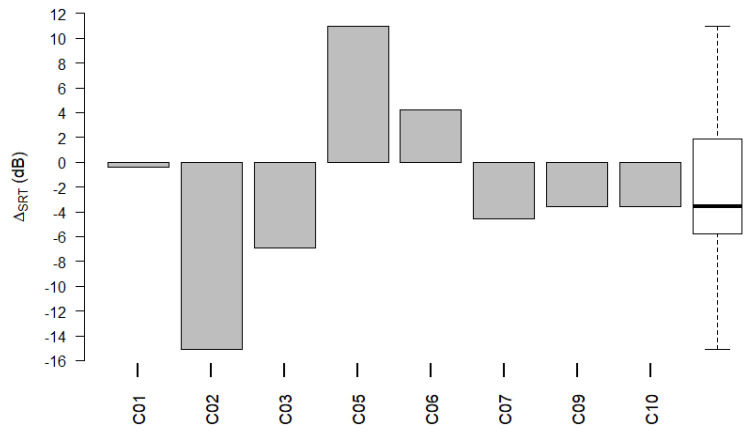
Δ_SRT_ per pediatric participant. The difference, Δ_SRT_, between RD_ecapSRT_ and RD_burstSRT_ for participants in the pediatric subgroup. Group data of Δ_SRT_ are shown as a boxplot to the right, where the median value is shown as a black horizontal line.

Figure 10 shows Δ_SRT_ for each participant of the pediatric subgroup. The SRTs of participant C08 when fitted with the LiveECAPMAP are not available because the LiveBurstMAP was accidentally saved on program locations 2 and 3. Therefore, participant C08 had to be excluded from the analysis of speech perception test scores.

The SRTs used in the analysis stem from a single OLKISA test list. RD_ecapSRT_ and RD_burstSRT_ did not differ significantly (Wilcoxon signed-rank test: *p* = 0.326). The mean Δ_SRT_ was −2.37 dB (95% c.i. −8.82 dB to +4.07 dB). In contrast to adults, the 2 dB criterion was not applied to the pediatric group, as was reflected in the Methods.

The absolute value of Δ_SRT_ was less than 2 dB only for participant C01. For all the other participants, the absolute value of Δ_SRT_ exceeded 2 dB. For participants C02 and C05, the absolute value of Δ_SRT_ exceeded 10 dB. Better results (lower SRTs) were achieved with the LiveECAPMAP in comparison to the LiveBurstMAP in participants C01, C02, C03, C07, C08, and C09. SRT achieved with LiveECAPMAP were better in comparison to the ClinMAP in participants C05, C07, and C09.

### 3.6. Adverse Events and Device Deficiencies

No adverse events and no device deficiencies were reported during or after the study.

## 4. Discussion

Currently, CI fitting requires a trained audiologist who sets the behavioral thresholds and MCL levels based on the subjective feedback of the person using the CI. This can be a challenging task when the person is not able to provide reliable (verbal) feedback. Objective measures such as the ECAP have been proposed to aid the fitting process in such cases [4,8].

The study presented in this paper evaluated the safety and performance of ARTFit in both adult and pediatric users of MEDEL CIs. Four evaluation measures were defined to assess ARTFit’s functionality in terms of the MCLs per channel of the electrode array, the average deviation of the fitting maps from the participants’ clinical map, fitting duration, and speech reception threshold. ARTFit uses ECAP thresholds to produce an initial fitting map. The ECAP-based initial fitting maps were compared to burst maps produced with standard behavioral procedures.

The relative difference between the MCL_ecap_ and the MCL_clin_ was found to not differ significantly (<20%) from the relative difference between the MCL_burst_ and the MCL_clin_. This, in turn, implies that the MCLs of the LiveECAPMAPs were non-inferior to the MCLs of the LiveBurstMAPs in both the adult and pediatric subgroups. Similarly, the magnitudes of the average deviation of the LiveECAPMAPs and the LiveBurstMAPs from the ClinMAPs were found to differ by less than 20%. In other words, the average deviation of the LiveECAPMAPs from the ClinMAPs was non-inferior to the average deviation of the LiveBurstMAPs from the ClinMAPs in both the adult and pediatric subgroups. The mean fitting duration of the LiveECAPMAPs was significantly shorter than that of the LiveBurstMAPs in both subgroups: 5.70 (range 1.90–11.98) vs. 9.27 (6.83–14.72) min in the adult subgroup and 3.03 (1.97–4.22) vs. 7.35 (3.95–12.77) min in the pediatric subgroup. The mean percentage difference, Δ_FD_, in the fitting duration between the LiveECAPMAP and the LiveBurstMAP was −37.5% in the adult and −50.4% in the pediatric subgroup.

The MCL_ecap_ of the LiveECAPMAPs did not exceed the comfortable loudness levels of our study participants. This is in line with the observation that none of the MCLs of the ECAPMAPs (before activation) exceeded the MCL_clin_. We can thus conclude that the ARTFit procedure does not lead to uncomfortable loudness or any painful sensations for the person using the CI. No adverse events were reported during or after the study, which demonstrates that ARTFit is a safe tool for producing initial fitting maps in the clinical setting.

Recently, it was suggested that the usefulness of objective measures such as the ECAP in fitting procedures should be assessed by measuring speech perception because speech perception is directly related to quality of hearing [8]. For the sake of the comparability of the data collected in this study, speech perception in the adult and pediatric subgroups was tested with OLKISA. Although OLKISA is suitable for ages ≥ 4 years, our experience in this study was that the test conditions were challenging for the pediatric participants. Speech perception tests were scheduled immediately after the fitting procedure. Some participants became easily distracted, physically active, tired, increasingly unresponsive, or impatient with the OLKISA procedure. Other participants were observed to perform well in the beginning but became less responsive towards the end of testing. There were some participants who spoke so softly that the test administrator could not be sure of their response. Hence, some of the poor scores that were recorded in this subgroup were not only due to a particular map but could also be due to other factors, such as the test procedure, the test environment, and attention span. Regarding individual participants, participants C02 and C05 were the youngest members of the pediatric subgroup in terms of both chronological age and hearing age (see Table 2). Participant C02 was fitted with a flat map instead of a LiveBurstMAP (see results on fitting duration), which might have led to a very high SRT score for this participant.

In a follow-up study, the authors might consider an additional test, such as the playful interactive Adaptive Auditory Speech Test (AAST) (Hörzentrum Oldenburg gGmbH, Oldenburg, Germany) [35]. The AAST is suitable for ages ≥ 4 years and has a similar test duration to OLKISA.

Having said that, conclusions can still be drawn about both subgroups’ overall performance in the speech perception tests. In both subgroups, the SRTs measured with the LiveECAPMAPs were non-inferior to those measured with the ClinMAPs. Similarly, the SRTs obtained with the LiveECAPMAPs were non-inferior to and did not significantly differ from the SRTs obtained with the LiveBurstMAPs. In other words, the participants’ performance in OLKISA when fitted with LiveECAPMAPs was comparable to their performance when they were fitted with the ClinMAPs and with the LiveBurstMAPs. The ECAP-based initial fitting maps produced with ARTFit did not compromise the study participants’ performance in the speech perception tests.

The map that resulted in the participants’ best individual performance differed from person to person. Averaging the SRT scores over all the participants in the adult and pediatric subgroups revealed that each subgroup’s overall best performance was with the ClinMAPs (see Table S1). This is not an unexpected outcome because the participants’ ClinMAPs are the result of adjustments to the map parameters that were made over several fitting sessions.

The correlation between the MCL_ecap_ and the MCL_clin_ across all channels was r^2^ = 0.732 (*p* < 0.001) in the adult subgroup and r^2^ = 0.616 (*p* < 0.001) in the pediatric subgroup. We compared these values with correlations that were calculated in other studies, i.e., the correlation between evoked stapedius reflex thresholds and behavioral MCLs and the correlation between maps based on the stapedius reflex threshold and behavioral MCLs. A high overall correlation of r^2^ = 0.846 (*p* < 0.001) between post-operative reflex thresholds and MCLs was found in a study with 6 adult participants who were fitted with MED-EL devices [36]. For all six participants, the median reflex thresholds and median MCLs had a similar dependence on the stimulated channels, which means the values of the median reflex threshold and median MCL recorded at each channel were similar, and this relationship was observed across all channels. Such a high correlation between post-operative reflex thresholds and the MCLs of behavioral maps suggests that the stapedius reflex threshold could be a useful objective measure for determining MCLs in adults. The usefulness of the reflex threshold was also assessed in a pediatric population. A correlation of r^2^ = 0.789 was found between MCLs that were calculated using the reflex threshold and MCLs that were measured with behavioral methods in 7 pediatric study participants who had at least 1 year of experience with their MED-EL devices [37]. The correlations we calculated between the MCL_ecap_ and the MCL_clin_ values were comparable to the correlations reported for the adult and pediatric populations of these two studies.

In our study, the correlation between the ECAP thresholds that were measured with ARTFit and the MCL_clin_ was r^2^ = 0.15 (*p* < 0.001) in the adult subgroup and r^2^ = 0.001 (*p* = 0.880) in the pediatric subgroup. Hence, only a weak significant correlation between ECAP thresholds and the MCLclin values was found in the adult subgroup, while a non-significant correlation was found in the pediatric subgroup. Recent studies that calculated correlations between ECAP thresholds and the MCLs of clinical maps obtained the following r^2^-values: 0.28 for 49 participants aged 1–67 years (mean age = 15 years) [4]; 0.14 for 47 pediatric participants [38]; 0.19 for 16 adult participants [14]; and 0.07 for 36 of 41 pediatric participants [39]. The values of r^2^ obtained in our study are thus comparable to these results and our study adds to the body of literature that report a weak correlation between ECAP thresholds and the MCLs of clinical maps. Furthermore, our results are in agreement with the recommendation made by Alvarez et al. [4] that ECAP thresholds can be used to calculate and set MCL levels in initial fitting maps.

Our study has its limitations, however. The aim of this clinical study was to assess the overall safety and performance of ARTFit. We designed the study to obtain and interpret group means in our four evaluation measures for both the adult and pediatric subgroups. We thus acknowledge that the individual results (see Table S1) obtained in both subgroups exhibit the between-subject variability in map parameters and in SRT scores that are well documented in the literature. Furthermore, our study is prone to selection bias due to the inclusion criteria applied to the study population.

The feasibility of ARTFit as a clinical tool for performing ECAP-based fittings is limited by two factors. Firstly, a weak correlation between the ECAP threshold and behavioral MCLs is well documented in the literature. Secondly, attempting to draw conclusions about the feasibility of ECAP-based fitting procedures from our results would necessarily be based on a group mean correlation between the ECAP threshold and behavioral MCL levels. The systematic literature review by de Vos et al. [8] recently evaluated study conclusions about ECAP-based fitting procedures. The authors found that most study conclusions are based solely on group mean correlations, which are not representative of the variability of individual within-subject correlations. There remains scope for a future follow-up study with ARTFit that takes between-subject variability into account.

The international survey conducted by Vaerenberg et al. [3] found that, in the absence of good clinical practice guidelines for behavioral CI fitting procedures, some common practices could still be identified. A single activation or switch-on session is usually followed by several fitting sessions during the first year. During follow-up sessions, several adjustments are made to the map parameters. These procedures are known to be quite effective in people who can provide reliable (verbal) feedback to the audiologist. In terms of its clinical application, ARTFit is useful for initial fittings in people who cannot provide subjective feedback.

Finally, a weak significant correlation between the ECAP thresholds that were measured with ARTFit and behavioral MCLs was found. It is thus important to note that although ARTFit can assist the audiologist with initial fittings, it cannot replace the need for behavioral feedback during or after activation sessions.

## 5. Conclusions

ARTFit is a safe, quick, and reliable tool for audiologists to produce ECAP-based initial fitting maps in adults and young children who cannot provide subjective feedback. It is suited for the task of estimating initial MCLs during activation (or switch-on) of the audio processor. The ECAP thresholds that were measured with ARTFit in this study did not correlate well with behavioral MCLs, which is a result well documented in the literature. ARTFit cannot replace the need for behavioral feedback during or after activation. During follow-up sessions, audiologists continue to adjust each person’s map parameters to their individual needs, according to subjective feedback.

## Figures and Tables

**Table 1:** Demographic data of the adult subgroup.

Subject ID ^1^	Age at Implantation (Years)	Duration of Hearing Loss (Years)	Age at Testing (Years)	Time of Hearing Loss ^2^	Implant ^3^	Electrode Array ^4^	Audio Processor	Disabled Channels ^5^
A02	43	43	46	Pre	SY	F28	SONNET	None
A03	47	Unknown	48	Pre	SY	F24	SONNET	E12
A04	66	25	69	Post	SY	STD	OPUS 2	None
A05	57	17	58	Post	SY	F28	SONNET	None
A06	59	1	62	Post	SY	F28	SONNET	None
A07	59	2	67	Post	SO	F24	OPUS 2	None
A08	64	57	68	Post	CO	F28	OPUS 2	None
A09	36	18	39	Post	SY	F28	SONNET	None
A10	58	2	65	Post	SO	STD	SONNET	None
A11	52	20	60	Post	SO	STD	OPUS 2XS	None
A12	64	51	69	Post	CO	FSO	SONNET	E01 & E02
A13	59	12	65	Post	CO	STD	SONNET	E11 & E12
A14	45	41	50	Post	CO	STD	OPUS 2	None
A15	20	14	32	Post	PU	STD	SONNET	None
A16	45	10	47	Post	SY	F28	SONNET	None
A17	66	3	68	Post	SY	F28	SONNET	None
A18	53	2	59	Post	CO	F28	SONNET	None
A19	63	5	67	Post	CO	F28	OPUS 2	None
A20	72	43	78	Post	CO	STD	OPUS 2	None
A21	52	13	56	Post	SY	F28	SONNET	None
A22	66	2	75	Post	SO	STD	SONNET	E11 & E12
A23	75	16	77	Post	SY	F24	RONDO	E12

^1^ Full subject ID starts with “ARTF_43_” followed by the displayed individual number (A = adult). ^2^ Pre = prelingual, Post = post-lingual. ^3^ SY = SYNCHRONY, SO = SONATA TI100, PU = PULSAR CI100, CO = CONCERTO. ^4^ STD = STANDARD, F28 = FLEX28, FSO = FLEXSOFT, F24 = FLEX24. ^5^ E refers to the number of the electrode channel.

**Table 2:** Demographic data of the pediatric subgroup.

Subject ID ^1^	Age at Implantation (Years)	Duration of Hearing Loss (Years)	Age at Testing (Years)	Time of Hearing Loss ^2^	Implant ^3^	Electrode Array ^4^	Audio Processor	Disabled Channels
C01	0.5	0.5	6	Pre	CO	F20	OPUS 2	None
C02	2.2	2.2	5	Pre	SY	F24	SONNET	None
C03	0.6	0.6	4	Pre	SY	F28	SONNET	None
C04	0.7	0.7	5	Pre	CO	F28	OPUS 2	None
C05	3.3	1.5	5	Pre	SY	F28	SONNET	None
C06	1.2	1.2	6	Pre	CO	F28	OPUS 2	None
C07	0.9	0.9	6	Con	CO	F24	OPUS 2	None
C08	0.8	0.8	7	Con	CO	F28	SONNET	None
C09	1.8	1.7	7	Pre	CO	F28	SONNET	None
C10	0.9	0.2	5	Pre	CO	F28	OPUS 2	None

^1^ Full subject ID starts with “ARTF_43_” followed by the displayed individual number (C = child). ^2^ Pre = prelingual, Con = congenital. ^3^ SY = SYNCHRONY, CO = CONCERTO. ^4^ F28 = FLEX28, F20 = FLEX20, F24 = FLEX24.

## Data Availability

All relevant data are within the manuscript and its supporting information file.

## Supplementary Materials

The following supporting information can be downloaded at: https://www.mdpi.com/article/10.3390/life12020269/s1, Table S1: Database. Maximum comfortable loudness (MCL) levels, fitting duration (FD), speech reception thresholds (SRTs), and speech coding strategies of the ClinMAP are listed for all participants.

## References

[1] Freni F., Gazia F., Slavutsky V., Scherdel E.P., Nicenboim L., Posada R., Portelli D., Galletti B., Galletti F. (2020). Cochlear implant surgery: Endomeatal approach versus posterior tympanotomy. Int. J. Environ. Res. Public Health..

[2] Lenarz T. (2018). Cochlear implant—State of the art. GMS Curr. Top Otorhinolaryngol. Head Neck Surg..

[3] Vaerenberg B., Smits C., De Ceulaer G., Zir E., Harman S., Jaspers N., Tam Y., Dillon M., Wesarg T., Martin-Bonniot D. (2014). Cochlear implant programming: A global survey on the state of the art. Sci. World J..

[4] Alvarez I., de la Torre A., Sainz M., Roldan C., Schoesser H., Spitzer P. (2010). Using evoked compound action potentials to assess activation of electrodes and predict C-Levels in the Tempo+ cochlear implant speech processor. Ear Hear..

[5] Sainz M., de la Torre A., Roldan C., Ruiz J.M., Vargas J.L. (2003). Analysis of programming maps and its application for balancing multichannel cochlear implants. Int. J. Audiol..

[6] Dawson P.W., Skok M., Clark G.M. (1997). The effect of loudness imbalance between electrodes in cochlear implant users. Ear Hear..

[7] Caldwell M.T., Jiam N.T., Limb C.J. (2017). Assessment and improvement of sound quality in cochlear implant users. Laryngoscope Investig. Otolaryngol..

[8] de Vos J.J., Biesheuvel J.D., Briaire J.J., Boot P.S., Van Gendt M.J., Dekkers O.M., Fiocco M., Frijns J.H.M. (2018). Use of electrically evoked compound action potentials for cochlear implant fitting: A systematic review. Ear Hear..

[9] Van Den Abbeele T., Noël-Petroff N., Akin I., Caner G., Olgun L., Guiraud J., Truy E., Attias J., Raveh E., Belgin E. (2012). Multicentre investigation on electrically evoked compound action potential and stapedius reflex: How do these objective measures relate to implant programming parameters?. Cochlear Implants Int..

[10] Gordon K., Papsin B.C., Harrison R.V. (2004). Programming cochlear implant stimulation levels in infants and children with a combination of objective measures. Int. J. Audiol..

[11] Kosaner J., Spitzer P., Bayguzina S., Gultekin M., Behar L.A. (2018). Comparing eSRT and eCAP measurements in pediatric MED-EL cochlear implant users. Cochlear Implant. Int..

[12] McKay C.M., Chandan K., Akhoun I., Siciliano C., Kluk K. (2013). Can ECAP measures be used for totally objective programming of cochlear implants?. J. Assoc. Res. Otolaryngol..

[13] Walkowiak A., Lorens A., Kostek B., Skarzynski H., Polak M. (2010). ESRT, ART, and MCL correlations in experienced paediatric cochlear Implant users. Cochlear Implant. Int..

[14] Walkowiak A., Lorens A., Polak M., Kostek B., Skarzynski H., Szkielkowska A., Skarzynski P.H. (2011). Evoked stapedius reflex and compound action potential thresholds versus most comfortable loudness level: Assessment of their relation for charge-based fitting strategies in implant users. ORL.

[15] He S., Teagle H.F.B., Buchman C.A. (2017). The electrically evoked compound action potential: From laboratory to clinic. Front. Neurosci..

[16] Lai W.K., Aksit M., Akdas F., Dillier N. (2004). Longitudinal behaviour of neural response telemetry (NRT) data and clinical implications. Int. J. Audiol..

[17] Botros A., van Dijk B., Killian M. (2007). AutoNRT™: An automated system that measures ECAP thresholds with the Nucleus Freedom cochlear implant via machine intelligence. Artif. Intell. Med..

[18] Gärtner L., Lenarz T., Joseph G., Büchner A. (2010). Clinical use of a system for the automated recording and analysis of electrically evoked compound action potentials (ECAPs) in cochlear implant patients. Acta Otolaryngol..

[19] Osterne F.J.V., Kós M.I., Botelho M., Félix F., Tomita S. ECAP measurements in the new MAESTRO 7.0: Comparison between AutoART and ART standard. Proceedings of the 15th International Conference on Cochlear Implants and Other Implantable Auditory Technologies.

[20] Botros A., Psarros C. (2010). Neural response telemetry reconsidered: I. The relevance of ECAP threshold profiles and scaled profiles to cochlear implant fitting. Ear Hear..

[21] Greisiger R., Shallop J.K., Hol P.K., Elle O.J., Jablonski G.E. (2015). Cochlear implantees: Analysis of behavioral and objective measures for a clinical population of various age groups. Cochlear Implant. Int..

[22] Van Dijk B., Botros A., Battmer R.D., Begall K., Dillier N., Hey M., Lai W.K., Lenarz T., Laszig R., Morsnowski A. (2007). Clinical results of AutoNRT, a completely automatic ECAP recording system for cochlear implants. Ear Hear..

[23] Cavalle-Garrido L., Schwarz K., Lauss K., de Paula-Vernetta C., Kontides A., Diaz-Gomez M., Guzman-Calvete A., Armengot-Carceller M. (2018). Comparison of a traditional and novel evoked compound action potentials recording approach and evoked auditory brainstem responses in pediatric cochlear implants users. J. Int. Adv. Otol..

[24] Gärtner L., Büchner A., Lenarz T., Schwarz K.E., Strahl S.B., Dierker A., Spitzer P. Evaluation of an automatic system to record and analyze electrically evoked compound action potentials. Proceedings of the 15th Symposium on Cochlear Implants in Children.

[25] Brown C.J., Hughes M.L., Luk B., Abbas P.J., Wolaver A., Gervais J. (2000). The relationship between EAP and EABR thresholds and levels used to program the Nucleus 24 speech processor: Data from adults. Ear Hear..

[26] Cullington H. (2000). Preliminary Neural Response Telemetry results. Br. J. Audiol..

[27] Smoorenburg G.F., Willeboer C., van Dijk J.E. (2002). Speech perception in Nucleus CI24M cochlear implant users with processor settings based on electrically evoked compound action potential thresholds. Audiol. Neurootol..

[28] Hughes M.L., Brown C.J., Abbas P.J., Wolaver A.A., Gervais J.P. (2000). Comparison of EAP thresholds with MAP levels in the Nucleus 24 cochlear implant: Data from children. Ear Hear..

[29] Strahl S.D., Spitzer P., Schwarz K. AutoART—A system for automatic determination of ECAP thresholds. Proceedings of the 21th annual Meeting of the German Society of Audiology (Deutsche Gesellschaft für Audiologie, DGA).

[30] Gärtner L., Lenarz T., Büchner A. (2018). Fine-grain recordings of the electrically evoked compound action potential amplitude growth function in cochlear implant recipients. Biomed. Eng. Online.

[31] Neumann K., Baumeister N., Baumann U., Sick U., Euler H.A., Weißgerber T. (2012). Speech audiometry in quiet with the Oldenburg Sentence Test for Children. Int. J. Audiol..

[32] Thomas J.P., Neumann K., Dazert S., Voelter C. (2017). Cochlear implantation in children with congenital single-sided deafness. Otol. Neurotol..

[33] (2021). Richtlinie des Gemeinsamen Bundesausschusses über die Verordnung von Hilfsmitteln in der vertragsärztlichen Versorgung. https://www.g-ba.de/richtlinien/13/.

[34] Van de Heyning P., Mertens G. Does a flat strategy based fitting map provide better or equal objective hearing performance as a single channel fitting map? In Proceedings of the 14th International Conference on cochlear implants and other Implantable Technologies, Toronto, ON, Canada, 11–14 May 2016.

[35] Hörzentrum Oldenburg gGmbH Adaptive Auditory Speech Test (AAST). https://www.hz-ol.de/de/diagnostik-aast.html.

[36] Stephan K., Welzl-Müller K. (2000). Post-operative stapedius reflex tests with simultaneous loudness scaling in patients supplied with cochlear implants. Audiology.

[37] Lorens A., Walkowiak A., Piotrowska A., Skarzynski H., Anderson I. (2004). ESRT and MCL correlations in experienced paediatric cochlear implant users. Cochlear Implant. Int..

[38] Muhaimeed H.A., Anazy F.A., Hamed O., Shubair E. (2010). Correlation between NRT measurement level and behavioral levels in pediatrics cochlear implant patients. Int J Pediatr Otorhinolaryngol..

[39] Holstad B.A., Sonneveldt V.G., Fears B.T., Davidson L.S., Aaron R.J., Richter M., Matusofsky M., Brenner C.A., Strube M.J., Skinner M.W. (2009). Relation of electrically evoked compound action potential thresholds to behavioral T-and C-levels in children with cochlear implants. Ear Hear..

